# Mutation of CarO participates in drug resistance in imipenem‐resistant *Acinetobacter baumannii*

**DOI:** 10.1002/jcla.22976

**Published:** 2019-07-18

**Authors:** Li‐Jing Zhu, Xiao‐Ying Chen, Pan‐Fei Hou

**Affiliations:** ^1^ Department of Clinical Laboratory Lianshui County People's Hospital Lianshui China; ^2^ Department of Clinical Laboratory, School of Medicine RenJi Hospital, Shanghai Jiao Tong University Shanghai China

**Keywords:** *Acinetobacter baumannii*, drug resistance, outer membrane proteins

## Abstract

**Objective:**

*Acinetobacter baumannii* has become an important problem because of the high drug resistance rate. The aim of this study was to assess the antimicrobial resistance profile and explore the role of membrane porin in imipenem resistance of *A baumannii*.

**Methods:**

A total of 63 isolates of imipenem‐resistant *A baumannii* (IRAB) and 21 of imipenem‐sensitive *A baumannii* (ISAB) were collected. Susceptibility testing to 16 kinds of antimicrobial agents was conducted by K‐B method. PCR technique was used to detect carO and oprD genes, and sequencing was performed to compare the sequence between IRAB and ISAB. Three‐dimensional structure model of CarO protein was established.

**Results:**

While ISAB isolates presented sensitive to most classes of antibiotics, isolates of IRAB displayed much higher resistance rate except tigecycline (3.2%), cefoperazone/sulbactam (28.6%), and minocycline (30.2%). All 84 isolates were observed carrying both carO and oprD genes. Further sequencing revealed important mutations of carO gene existed in IRAB in comparison with ISAB. Meanwhile, significant differences in three‐dimensional structure of carO protein molecule were also found between IRAB and ISAB.

**Conclusions:**

The drug resistance profile of IRAB is increasingly severe in clinical settings. Mutation of CarO was identified as one of the molecular mechanisms involved in imipenem resistance in *A baumannii*.

## 1 INTRODUCTION

*Acinetobacter baumannii*, a nonfermentative, gram‐negative coccobacillus, has emerged as a ubiquitous opportunistic pathogen leading to nosocomial infections with high morbidity and mortality.^1^ It mainly causes respiratory tract infection, as well as bacteremia, surgical site infection, digestive system, urinary tract infection, etc Imipenem has been frequently used to treat *A baumannii* infection due to both effective antibacterial activity and less frequent side effects. However, the increasing emergence of IRAB worldwide has created severe challenges to therapeutic strategies.^2^ It is significant to explore the resistance mechanism of *A baumannii* to imipenem.

The molecular mechanism of drug resistance includes enzymatic hydrolysis such as carbapenemase, the overexpression of active efflux pump, and reduced permeability as a result of outer membrane proteins OMPs) loss or modification.^3^ Carbapenemase was considered the main cause of imipenem resistance in the previous study, although it was reported that there was no carbapenemase detected in some IRAB.^4^ The OMPs have been a hot research topic in recent years. The major OMPs associated with imipenem resistance include CarO and OprD.^5^ CarO protein, generally considered as an eight‐stranded β‐barrel protein of 29 KDa,^6^ plays a key role in the influx of imipenem (but not meropenem) into *A baumannii*. It could be divided into two groups, CarOa and CarOb, in which CarOb was showed to be twice more specific for imipenem than CarOa.^7^ Mutations in the carO gene would alter the structure, decrease or delete the expression of the porin, resulting in reduction of antibiotics entry into the bacteria. OprD, a 43 KDa porin, is the main and specific porin for uptake of carbapenems into *A baumannii*.^8^ *A baumannii* also possesses an OprD homologue, which is normally considered to be involved in carbapenems resistance.^9^, ^10^ However, a few studies suggested that OprD homologue in *A baumannii* does not really associate with resistance to carbapenem.^11^, ^12^

The role of CarO and OprD in *A baumannii* is still in controversy. This study aims to assess the antimicrobial resistance profile and explore the role of CarO and OprD in imipenem resistance of *A baumannii*.

## 2 MATERIALS AND METHODS

### 2.1 Bacterial isolates

From January to June 2016, a total of 84 nonduplicate isolates of *A baumannii*, including 63 IRAB and 21 ISAB, were randomly collected from many kinds of specimens such as sputum, pus, drainage fluid, urine, blood, and pharyngeal swabs in Shanxi Dayi Hospital, a teaching hospital with over 1000 beds. All strains selected were confirmed by Vitek2 compact system (bioMerieux Co., French). The strain of *A baumannii* ATCC25922 purchased from the National Center of Clinical Laboratory served as a control.

### 2.2 Antimicrobial susceptibility testing

The resistance phenotype was obtained by K‐B method for the following 16 antibiotics: ampicillin/sulbactam, cefazolin, cefoxitin, ceftriaxone, cefepime, cefoperazone/sulbactam, imipenem, meropenem, gentamicin, tobramycin, amikacin, minocycline, tigecycline, ciprofloxacin, levofloxacin, and trimethoprim/sulfamethoxazole, which were all purchased from OXOID Company. Specific protocols and interpretation criteria get from the Clinical and Laboratory Standards Institute CLSI), 2016.^13^ The results were analyzed with WHONT software.

### 2.3 Detection of carO and oprD genes

Genomic DNA was extracted by boiling method. Primers were produced by Invitrogen Ltd. Co., Shanghai, China. Sequences of primers (5′‐3′) were as follows: carO P1: ATGAAAGTATTACGTGTTTTAGTGACAAC, P2: TTACCAGTAGAATTCTACACCAACT; oprD P1: ATGCTAAAAGCACAAAAACTTACATTAGCA, P2: TTAGAATAATTTCACAGGAATATCTAAGAA. PCR Kit was purchased from Takara Bio, Dalian, China. PCR amplification was performed with a 25 µL reaction mixture according to the kit instructions. The cycling protocol was conducted on PCR thermal cycler machine (ABI 7500; Life Technologies Co) as follows: an initial denaturation step at 94°C for 5 minutes, followed by 30 cycles of 30 seconds at 94°C, 45 seconds at 55°C and 1 minute at 72°C, and finally finished with 5 minutes at 72°C. PCR products were assessed by 1% agarose gel electrophoresis. The specific amplification bands predicted about 729 bp and 1320 bp for carO and oprD, respectively, were observed with the gel imager.

### 2.4 Sequencing analysis

PCR products from both IRAB and ISAB isolates were sequenced by Invitrogen Ltd. Co., Shanghai, China. The carO and oprD gene sequences were analyzed by DNASTAR software. Additionally, amino acid sequences translated from these gene sequences were also analyzed.

### 2.5 Three‐dimensional structural modeling of CarO protein

CarO amino acid sequences from one strain of IRAB and one strain of ISAB were submitted to SWISS‐MODEL workspace for three‐dimensional structure modeling (PBD:4rlb as a template), and the similarity analysis was performed by molecular visual software Cn3D.

## 3 RESULTS

### 3.1 Antimicrobial susceptibility patterns

The susceptibility profiles of total 84 isolates are presented in Table 1. IRAB exhibited the lowest resistance rate to tigecycline (3.2%), followed by cefoperazone/sulbactam (28.6%) and minocycline (30.2%). For all the remaining antibiotics included in the table, IRAB possessed resistance rate greater than 70%. In comparison, ISAB strains showed a wider range of susceptible agents except ampicillin/sulbactam, cefazolin, and cefoxitin.

**Table 1:** Antimicrobial susceptibility profiles of IRAB and ISAB [n(%)]

Antibiotics	IRAB (n = 63)			ISAB (n = 21)		
	*R*	*M*	*S*	*R*	*M*	*S*
Ampicillin/sulbactam	63 (100)	0 (0)	0 (0)	21 (100)	0 (0)	0 (0)
Cefazolin	63 (100)	0 (0)	0 (0)	21 (100)	0 (0)	0 (0)
Cefoxitin	63 (100)	0 (0)	0 (0)	21 (100)	0 (0)	0 (0)
Ceftriaxone	63 (100)	0 (0)	0 (0)	0 (0)	16 (76.2)	5 (23.8)
Cefepime	63 (100)	0 (0)	0 (0)	4 (19.0)	1 (4.8)	16 (76.2)
Cefoperazone/sulbactam	18 (28.6)	39 (61.9)	6 (9.5)	2 (9.5)	2 (9.5)	17 (81)
Imipenem	63 (100)	0 (0)	0 (0)	0 (0)	0 (0)	21 (100)
Meropenem	63 (100)	0 (0)	0 (0)	1 (4.8)	0 (0)	20 (95.2)
Gentamicin	59 (93.6)	1 (1.6)	3 (4.8)	1 (4.8)	0 (0)	20 (95.2)
Tobramycin	46 (73.0)	1 (1.6)	16 (25.4)	0 (0)	0 (0)	21 (100)
Amikacin	49 (77.8)	0 (0)	14 (22.2)	3 (14.3)	2 (9.5)	16 (76.2)
Minocycline	19 (30.2)	40 (63.5)	4 (6.3)	0 (0)	2 (9.5)	19 (90.5)
Tigecycline	2 (3.2)	15 (23.8)	46 (73.0)	0 (0)	0 (0)	21 (100)
Ciprofloxacin	63 (100)	0 (0)	0 (0)	11 (52.4)	6 (28.6)	4 (19)
Levofloxacin	47 (74.6)	16 (25.4)	0 (0)	7 (33.3)	2 (9.5)	12 (57.2)
Trimethoprim/sulfamethoxazole	49 (77.8)	0 (0)	14 (22.2)	3 (14.3)	3 (14.3)	15 (71.4)

Abbreviations: IRAB, imipenem‐resistant *Acinetobacter baumannii*; ISAB, imipenem‐sensitive *Acinetobacter baumannii*; *M*: mediate; *R*: resistant; *S*: sensitive.

### 3.2 Detection of OMPs genes

**Figure 1:**
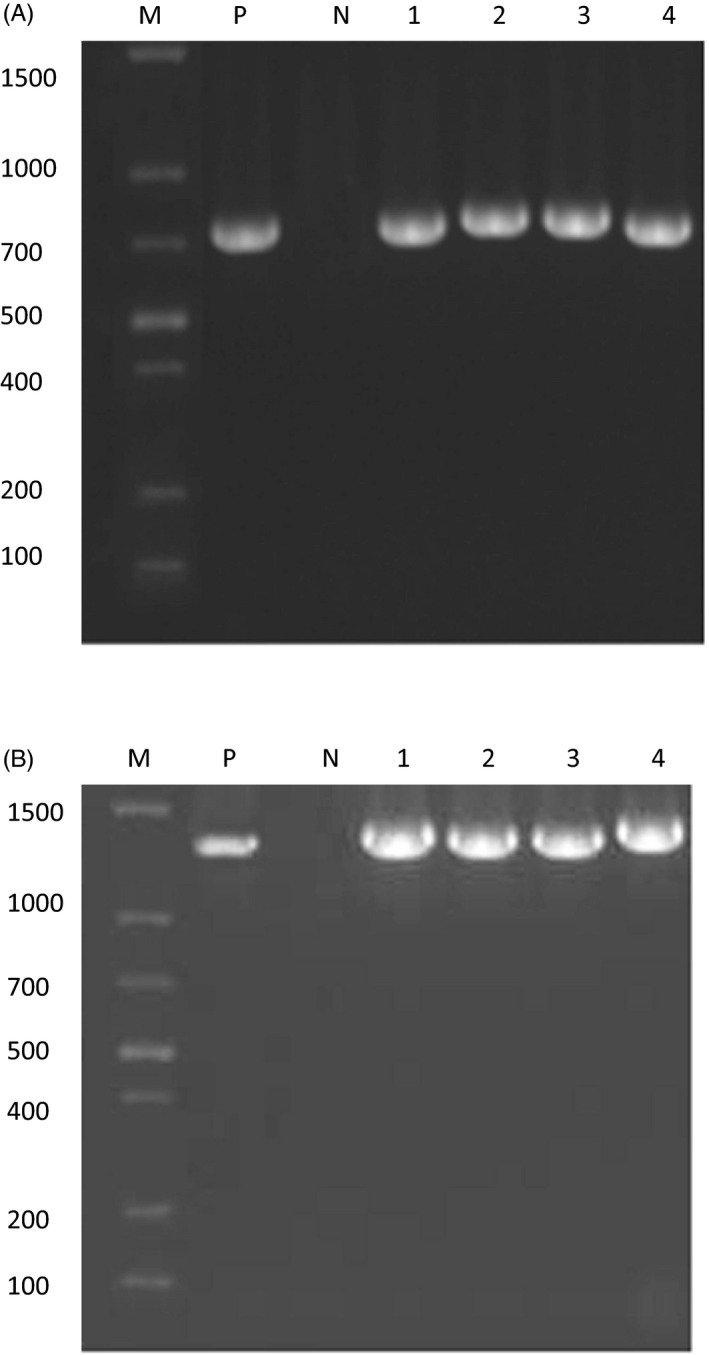
Detection of carO and oprD genes by PCR. A, 1‐4: oprD genes; B, 1‐4: carO genes. M: molecular weight marker; N: negative control; P: positive control

All 84 isolates were observed carrying both carO and oprD genes. Electrophoresis of these genes is shown in Figure 1. Further sequence alignment revealed important mutations of carO gene existed in IRAB in comparison with ISAB, while no significant mutations of oprD gene were found.

### 3.3 Three‐dimensional structural modeling

**Figure 2:**
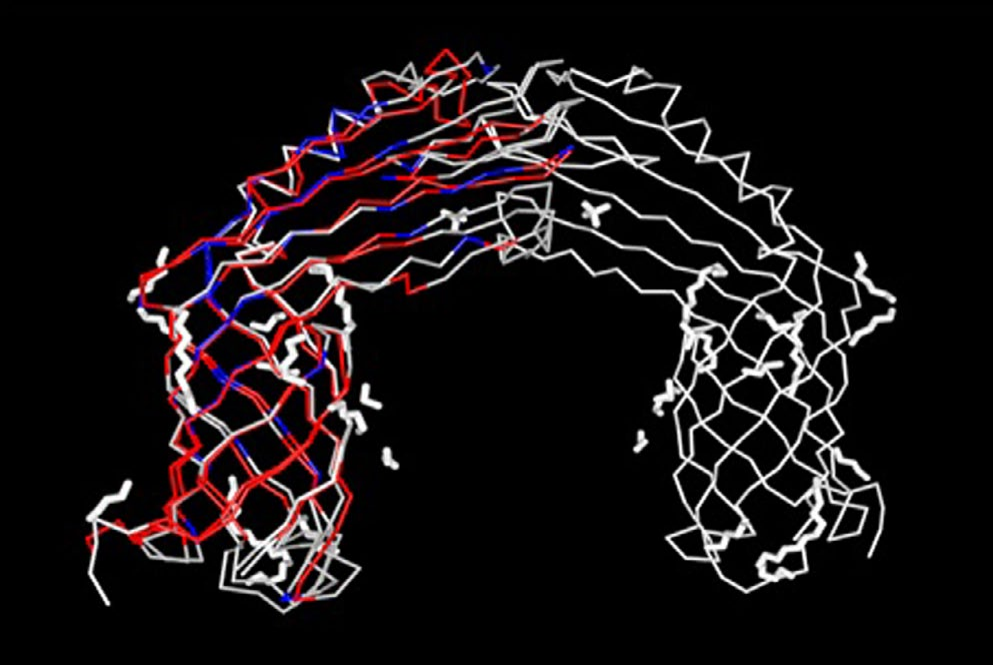
Three‐dimensional Structure of CarO protein. Red: same amino acids; blue: different amino acids; gray: unmatched sequences

Amino acid sequences translated from carO gene sequences exhibited about 80.9% similarity coefficient between IRAB and ISAB. The main differences included amino acids deletion at position 133, as well as insertion at 140‐141 and 154‐156. However, oprD in IRAB only showed one difference at the 48th position (threonine → serine), which has little relationship with antibiotic resistance. Furthermore, significant differences in three‐dimensional structure of carO protein were found between IRAB and ISAB (Figure 2).

## 4 DISCUSSION

*Acinetobacter baumannii*, which widely exists in natural soil, water, hospital environment and human skin, urogenital tract, digestive tract, and respiratory tract, is an important pathogen of nosocomial infection.^14^ Along with the increasing infection caused by *A baumannii*, antibiotic resistance is also on the rise in recent years. The resistance rate of *A baumannii* to imipenem in China increased from 31.0% to 62.4% in recent 10 years.^15^ Resistance against carbapenems is sufficient to define an *A baumannii* as highly resistant, since it often presents multi‐drug, pan‐drug, and even full‐drug resistance.^16^, ^17^

In this study, IRAB displayed high resistance to most common antibiotics except tigecycline, minocycline, and cefoperazone/sulbactam, consisting of previous reports.^18^ Therefore, powerful prevention and control of nosocomial infection is particularly important to avoid the situation of no medicine available. First, clinical management of the use of antibiotics should be strengthened to slow or reduce the emergence of drug resistance. Additionally, aseptic operation and disinfection isolation system should be strictly implemented to prevent the spread of drug‐resistant bacteria.

Decreased expression of the OMPs was significantly associated with carbapenem resistance.^3^ However, the relationship between CarO or OprD mutation and imipenem resistance has been the focus of controversy. It is reported in several studies that CarO and OprD participate in the resistance of imipenem with nonspecific and specific monomeric channel in *A baumannii*, respectively.^6^, ^9^, ^19^ However, a research in Brazil showed that OXA‐23 carbapenemase was the major carbapenem resistance mechanism and loss of CarO porin plays a minor role in this phenotype.^20^ Zhang YP et al reported that no significant change was found in the expression of oprD and carO in IRAB.^21^ Our results revealed that all 84 isolates included in this study possess both carO and oprD genes. Comparing amino acid sequences, OprD from IRAB and ISAB only exhibited one difference at the 48th position (threonine → serine, both polar amino acid, Figure S1), which has little relationship with antibiotic resistance. Concerning CarO, 80.9% concordance of amino acids was found between IRAB and ISAB. Three‐dimensional structural modeling showed that significant modifications such as deletion, insertion, or polarity reversal of CarO amino acids mostly occurred at the position of β folds. Conformational changes in porin CarO caused by carO gene mutations eventually reduce the permeability of outer membrane and lead to drug resistance. This result is consistent with the reports of Benmahmodet al,^12^ but different from that of Moran‐Barrio J et al^10^ It may be due to different epidemic types of the bacteria and multiple drug resistance mechanisms involved in different regions. Further experiments such as quantitative analysis of OMPs mRNA expression and structural and functional research of OMPs between IRAB and ISAB are necessary for the future.

In summary, the resistance profile of IRAB is increasingly severe in clinical settings. Conformation change in CarO porin was identified as one of the important molecular mechanisms involved in imipenem resistance in *A baumannii*.
