# Supplementary figures and images for: Mutation of CarO participates in drug resistance in imipenem‐resistant Acinetobacter baumannii
Source: J Clin Lab Anal. 2019 Jul 18;33(8):e22976. doi: 10.1002/jcla.22976 (PMC6805298; doi:10.1002/jcla.22976)

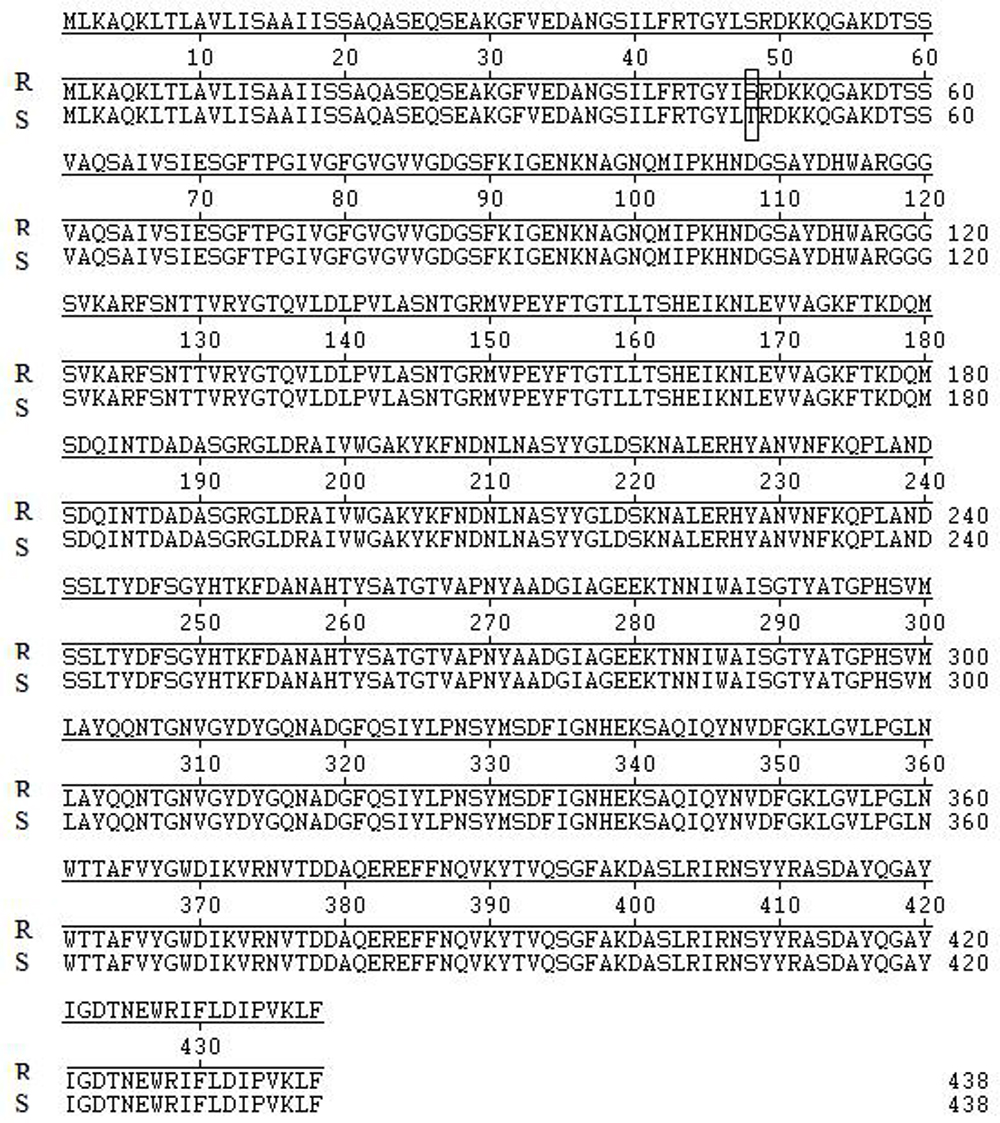

Supplement: Supplementary file 1 [file JCLA-33-e22976-s001.jpg]
